# Effects of inner ear abnormalities on middle ear mechanics: Findings from adults with MD and LVAS

**DOI:** 10.1016/j.bjorl.2025.101673

**Published:** 2025-09-27

**Authors:** Wen Jiang, Yi Mu, Huan Lin, Chanfeng Shen, Huiying Zhang, Fei Zhao, Yuehua Qiao, Xuanyi Li, Wen Liu

**Affiliations:** a The First College of Clinical Medicine, Xuzhou Medical University, Xuzhou, China; b Department of Otolaryngology, The Affiliated Hospital of Xuzhou Medical University, Xuzhou, China; c Auditory Engineering Laboratory of Jiangsu Province, The Second College of Clinical Medicine, Xuzhou Medical University, Xuzhou, China; d The College of Medical Technology, Xuzhou Medical University, Xuzhou, China; e Centre for SLT and Hearing Sciences, Cardiff School of Sport and Health Sciences, Cardiff Metropolitan University, Cardiff CF5 2YB, Wales

**Keywords:** Third window, Wideband acoustic immittance, MD, LVAS

## Abstract

**Objective:**

To evaluate the clinical significance of WAI as a predictive marker for MD and LVAS among adults.

**Methods:**

Participants included 110 ears with MD, 18 ears with LVAS, and 92 normal ears (control group) recruited from clinical audiology settings. The outcome measures included Wideband Energy Absorbance (WBA), Resonance Frequency (RF), admittance Magnitude (YM), and phase Angle (YA). Propensity Score Matching (PSM) was conducted to treat the group imbalance.

**Results:**

Compared with the control group, The MD group exhibited significantly lower WBA between 1587 and 4000 Hz (p < 0.05), while the LVAS group showed lower WBA between 1000 and 2520 Hz (p < 0.05). The MD and LVAS groups had lower RF (p < 0.05), while they had higher YM at low frequencies (226 and 678 Hz for MD, and 1000 Hz for LVAS, p < 0.05).

**Conclusions:**

This study showed that LVAS has a lower WBA at middle frequencies, whereas MD has a lower WBA at middle and high frequencies. These findings suggest that WAI may serve as a predictive marker for MD and LVAS. However, further studies are needed to explore its diagnostic utility in Third Mobile Window Abnormalities (TMWA).

**Level of evidence:**

IV: Retrospective cohort study.

## Highlights

• Unique WAI patterns reveal middle ear mechanics differences in MD and LVAS.
• WAI differentiates MD and LVAS via WBA changes across frequencies.
• VA imaging may improve WAI's use in diagnosing inner ear issues.

## Introduction

Inner ear disorders such as Meniere’s Disease (MD) and Large Vestibular Aqueduct Syndrome (LVAS) not only affect inner ear fluid dynamics but may also significantly alter middle ear sound transmission by impacting the mechanics involved in sound energy transfer.^1^, ^2^, ^3^ These conditions contribute to conductive hearing loss due to Third Mobile Window Abnormalities (TMWA), where abnormal inner ear openings alter intracochlear pressure dynamics and modify impedance mismatch between the middle and inner ear.^2^ These altered pressure dynamics complicate the diagnosis and treatment of conditions that may initially appear as typical middle ear disorders. For instance, Air-Bone Gaps (ABGs) observed in audiograms are typically associated with middle ear pathologies but may also occur in patients with MD and LVAS.^4^

Unlike Superior Semicircular Canal Dehiscence (SSCD), which leads to decreased inner ear impedance, MD and LVAS are characterized by increased inner ear pressure, affecting sound transmission differently.^3^ This difference in pressure dynamics leads to distinct effects on sound transmission through the middle ear. MD is characterized by endolymphatic hydrops, where excess fluid accumulates in the inner ear, while LVAS involves an enlarged vestibular aqueduct, creating a third window effect that disrupts sound energy transmission.^3^ Understanding the impact of this increased inner ear pressure on middle ear mechanics in both conditions is crucial for elucidating the underlying mechanisms of sound energy transmission and its subsequent disruptions.

Wideband Acoustic Immittance (WAI) has emerged as a promising tool for assessing middle ear function across a wide frequency range, offering a more detailed analysis than traditional single-frequency tympanometry.^3^, ^4^ Studies have shown that WAI is effective in detecting various middle ear pathologies, such as otitis media, ossicular chain disruption, and otosclerosis, suggesting its potential to identify the mechanical disruptions seen in MD and LVAS.^4^, ^5^, ^6^

This study aims to evaluate the clinical utility of WAI in adults diagnosed with LVAS or MD, focusing on its ability to serve as a predictive biomarker for these inner ear abnormalities. We hypothesize that WAI will reveal distinct patterns of Wideband Absorbance (WBA), resonant frequencies, admittance magnitude, and phase angle in LVAS and MD patients, compared to a normal control group. By exploring these patterns, this study seeks to deepen the understanding of how increased inner ear pressure affects middle ear mechanics and to identify potential diagnostic markers for these conditions.

## Methods

### Study participants

The MD group comprised 95 adults (42 males, 53 females) recruited from clinical audiology settings between January 2018 and October 2022. Inclusion criteria required a diagnosis of MD based on established clinical guidelines, which included a history of at least two vertigo episodes lasting between 20 min and 12 h, fluctuating hearing loss, and either tinnitus or aural fullness in the affected ear.^7^, ^8^ Additionally, all participants exhibited endolymphatic hydrops on Gadolinium-enhanced MRI. Individuals with significant middle-ear pathology or abnormal findings on otoscopy were excluded. Tympanometry was conducted at 226 Hz, and only participants with a Type A tympanogram were included in the analysis. Among the MD group, 15 participants presented with bilateral MD, resulting in a total evaluation of 58 left ears and 52 right ears.

The LVAS group consisted of 9 adults (8 males, 1 female). Eligibility for this group required the absence of significant middle-ear pathology, confirmed by otoscopy, which showed a clean external auditory canal and a healthy tympanic membrane. Hearing loss was confirmed through pure-tone audiometry. A Type A tympanogram at 226 Hz with normal external auditory canal volume was mandatory for inclusion. Bilateral large vestibular aqueducts, defined as ≥1.5 mm on CT imaging, were also required for group membership.

The control group included 46 normal-hearing adults (92 ears; 35 males, 57 females) who volunteered for the study. Participants were screened to ensure normal hearing, defined as pure-tone audiometry thresholds ≤20 dB HL across frequencies from 0.25 to 8 kHz with no air-bone gap. Participants had no significant history of middle-ear pathology or aural symptoms. Otoscopy confirmed a clean external auditory canal and a healthy tympanic membrane. Tympanometry was performed at 226 Hz, and only those with a Type A tympanogram and normal external auditory canal volume were included.

### Clinical methods

This study was conducted in accordance with Good Clinical Practice and the ethical principles outlined in the Declaration of Helsinki. Approval was obtained from the Ethics Committee. Due to the retrospective nature of the study, a waiver of informed consent was granted. Comprehensive audiological assessments were completed prior to the experimental evaluation of WAI.

WAI measurements were conducted using the Interacoustics IMP440 system (Interacoustics, Denmark). This system was employed to evaluate the mechanical properties of the middle ear across a frequency range of 226 Hz–8000 Hz and a pressure range from −300 daPa to +200 daPa. These parameters allow for a comprehensive assessment of the middle ear's function, capturing data that reflect its response to a broad spectrum of sound frequencies and varying air pressures.

To ensure the precision of the data collected, silicone-based probe tips of various sizes were utilized to create a secure seal within the ear canal. The system performed an automatic seal check to confirm the absence of air leakage before measurements commenced. The tests were conducted in a quiet, controlled environment, with participants instructed to remain as still as possible to minimize movement artifacts, which could otherwise distort the results.

The WAI system automatically calculated the Resonance Frequency (RF), defined as the lowest frequency at which the susceptance (B) reaches zero mmhos. This frequency indicates the point where the mass and stiffness components of the middle ear are in equilibrium, enabling optimal sound energy transfer. Additionally, the system recorded WBA, admittance magnitude, and phase angle. For analysis, only measurements taken at tympanometric peak pressure was considered.

### Statistical analysis

To minimize selection bias, Propensity Score Matching (PSM) was implemented to balance the groups. The matching process aimed for a negligible difference between groups, as indicated by an absolute standardized mean difference (*d*) of less than 0.1. Matching was performed using *R* software (version 4.2.2), with a 1:1 matching ratio and a caliper value of 0.2. The Shapiro-Wilk test was utilized to assess the normality of the data. Categorical variables were presented as numbers and percentages, while quantitative comparisons between two groups were made using either the Student’s *t*-test or the Mann-Whitney *U* test, depending on the distribution of the data.

## Results

### Participant characteristics

This study included a total of 150 adults, corresponding to 220 ears (Table 1, Table 2, Supplement Table S1). As audiometric tests were conducted at the ear level, the analyses were performed on individual ears rather than on the participants as a whole. Age was significantly imbalanced between the MD and control groups prior to matching (pre-matched p < 0.05, Table 1), and gender showed an imbalance between the LVAS and control groups (pre-matched p < 0.001, Table 2).

**Table 1:** Baseline Covariates between the control and MD groups with PSM.

Baseline Covariate	Pre-Propensity - Matching		p-value	Post-Propensity - Matching		p-value
	Control (n = 92)	MD (n = 110)		Control (n = 41)	MD (n = 41)	
Age (yr.)	30.89 ± 17.16	51.85 ± 12.74	<0.001	43.49 ± 13.59	43.46 ± 13.04	0.99
Gender			0.36			1.00
Male	35 (38.0%)	50 (45.5%)		22 (53.7%)	21 (51.2%)	
Female	57 (62.0%)	60 (54.5%)		19 (46.3%)	20 (48.8%)	
Ear side			0.81			0.83
Left	46 (50.0%)	58 (52.7%)		21 (51.2%)	23 (56.1%)	
Right	46 (50.0%)	52 (47.3%)		20 (48.8%)	18 (43.9%)

**Table 2:** Baseline Covariates between the control and LVAS groups with PSM.

Parameter	Pre-Propensity - Matching			Post-Propensity - Matching		
	Control (n = 92)	LVAS (n = 18)	p-value	Control (n = 18)	LVAS (n = 18)	p-value
Age (years)	30.89 ± 13.56	27.44 ± 4.66	0.326	27.56 ± 4.68	27.44 ± 4.66	0.94
Gender			<0.001			1.00
Male	35 (38.0%)	16 (88.9%)		16 (88.9%)	16 (88.9%)	
Female	57 (62.0%)	2 (11.1%)		2 (11.1%)	2 (11.1%)	
Ear side			1.00			1.00
Left	46 (50.0%)	9 (50.0)		9 (50%)	9 (50%)	
Right	46 (50.0%)	9 (50.0%)		9 (50%)	9 (50%)	

**Fig. 1:**
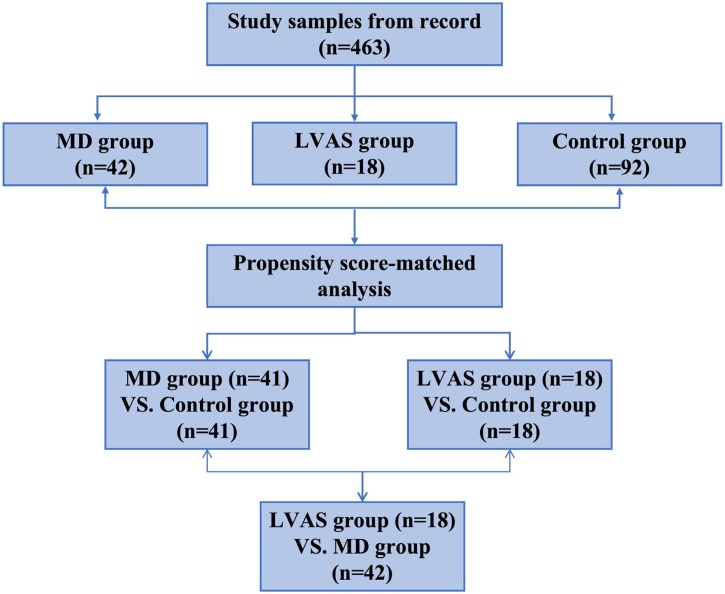
Flow chart of study population selection.

To address these baseline imbalances in age and gender, Propensity Score Matching (PSM) was applied, following the method described by Staffa et al.^9^ After matching, the absolute standardized mean difference for each variable was *d* < 0.1, indicating a successful balance between the groups. For the final analysis, 41 ears from the MD group and 18 ears from the LVAS group were matched with controls, ensuring covariate equivalence in terms of age, gender, and ear side (Fig. 1).

While PSM was applied to balance the groups in terms of age and gender, achieving perfect audiometric matching was not feasible due to the limited number of LVAS cases available for analysis. All LVAS participants exhibited profound hearing loss (>70 dB HL), whereas the MD group had a broader range of hearing loss from mild to severe (25–70 dB HL) (Supplement Table S1). Due to systematic difference in auditory profiles, strict hearing threshold matching would have substantially reduced the sample size, thereby compromising statistical power and generalizability. Given the rarity of LVAS, an exact audiometric match was impractical, but we ensured transparency by providing audiometric distributions and discussing their potential influence on WAI findings.

### Wideband Absorbance (WBA) in adults with MD and LVAS

**Fig. 2:**
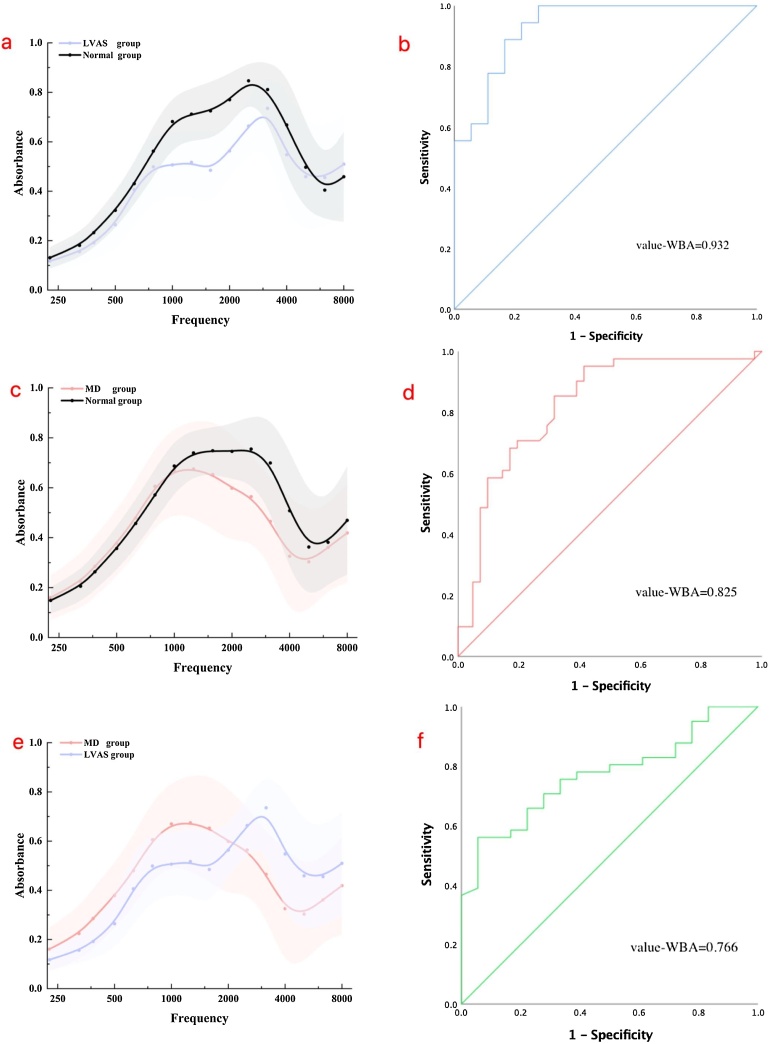
Comparison of absorbance at peak pressure between the LVAS, MD and control groups, the means (± Standard Deviation of the mean) are shown in colored lines, with shaded regions representing the standard error. ROC curves are included for each comparison. The shaded region indicates the range of mean ± 1 Standard Deviation (SD) across the frequencies. between 226 and 8000 Hz.

The LVAS group exhibited significantly lower WBA compared to the control group at frequencies between 1000 and 2520 Hz (p < 0.05; Fig. 2a). The ROC curve for distinguishing between the LVAS and control groups is shown in Fig. 2b, with a ROC value of 0.932. In the MD group, WBA was significantly lower than in the control group at frequencies between 2000 and 4000 Hz (p < 0.05; Fig. 2c). The ROC curve for distinguishing between the MD and control groups is shown in Fig. 2d, with a ROC value of 0.825. WBA differed significantly between the MD and LVAS groups at 1000–1260 Hz and 3175–4000 Hz (p < 0.05; Table 3). Both groups exhibited typical WBA curves, as illustrated in Fig. 2e, with a ROC value of 0.766 for differentiating between MD and LVAS (Fig. 2f).

**Table 3:** Comparison of RF, WBA, YM and YA under peak pressure among MD, LVAS and Control groups.

Variables (Hz)	Control A (n = 41)	Control B (n = 18)	MD (n = 41)	LVAS (n = 18)	P1	P2	P3
RF	850.61 ± 236.90	943.61 ± 111.36	748.00 ± 223.80	794.50 ± 172.23	**0.047**	**0.004**	0.436
WBA							
226	0.15 ± 0.05	0.13 ± 0.04	0.16 ± 0.09	0.12 ± 0.04	0.484	0.346	**0.014**
324	0.21 ± 0.06	0.18 ± 0.05	0.22 ± 0.11	0.16 ± 0.05	0.347	0.160	**0.014**
386	0.26 ± 0.07	0.23 ± 0.06	0.28 ± 0.12	0.19 ± 0.07	0.332	0.067	**0.004**
500	0.36 ± 0.09	0.32 ± 0.08	0.38 ± 0.13	0.26 ± 0.11	0.402	0.064	**0.002**
630	0.46 ± 0.10	0.43 ± 0.08	0.48 ± 0.14	0.41 ± 0.15	0.414	0.544	0.080
794	0.57 ± 0.10	0.56 ± 0.11	0.61 ± 0.16	0.50 ± 0.14	0.248	0.132	**0.016**
1000	0.69 ± 0.11	0.68 ± 0.10	0.67 ± 0.17	0.51 ± 0.12	0.590	**<0.001**	**<0.001**
1260	0.74 ± 0.09	0.71 ± 0.10	0.67 ± 0.19	0.52 ± 0.14	0.057	**<0.001**	**0.003**
1587	0.75 ± 0.09	0.73 ± 0.12	0.65 ± 0.22	0.48 ± 0.15	**0.012**	**<0.001**	**0.004**
2000	0.75 ± 0.11	0.77 ± 0.11	0.60 ± 0.21	0.56 ± 0.10	**<0.001**	**<0.001**	0.391
2520	0.75 ± 0.13	0.85 ± 0.07	0.56 ± 0.17	0.66 ± 0.11	**<0.001**	**<0.001**	**0.030**
3175	0.70 ± 0.18	0.81 ± 0.12	0.46 ± 0.17	0.74 ± 0.15	**<0.001**	0.092	**<0.001**
4000	0.51 ± 0.24	0.67 ± 0.21	0.33 ± 0.24	0.55 ± 0.26	**<0.001**	0.140	**0.002**
5040	0.36 ± 0.21	0.50 ± 0.18	0.30 ± 0.20	0.46 ± 0.22	0.192	0.566	**0.009**
6350	0.38 ± 0.17	0.40 ± 0.12	0.36 ± 0.17	0.46 ± 0.20	0.577	0.366	0.067
8000	0.47 ± 0.22	0.46 ± 0.18	0.42 ± 0.20	0.51 ± 0.21	0.276	0.439	0.116
YM							
226	2.15 ± 0.40	1.98 ± 0.40	2.42 ± 0.57	2.04 ± 0.37	**0.012**	0.626	**0.010**
678	5.70 ± 1.03	5.41 ± 1.22	6.47 ± 1.51	5.18 ± 1.10	**0.009**	0.562	**0.002**
800	6.71 ± 1.18	6.32 ± 1.56	7.37 ± 1.79	5.58 ± 1.27	0.053	0.128	**<0.001**
1000	7.80 ± 1.28	7.09 ± 1.57	8.19 ± 2.20	5.99 ± 1.28	0.333	**0.027**	**<0.001**
YA							
226	1.37 ± 0.06	1.38 ± 0.05	1.37 ± 0.09	1.44 ± 0.18	0.768	0.142	**0.040**
678	1.13 ± 0.09	1.14 ± 0.09	1.10 ± 0.20	1.51 ± 1.16	0.372	0.176	**0.029**
800	1.06 ± 0.13	1.05 ± 0.13	1.01 ± 0.22	1.53 ± 1.35	0.194	0.145	**0.020**
1000	0.92 ± 0.17	0.89 ± 0.17	0.90 ± 0.30	1.61 ± 1.67	0.759	0.077	**0.010**

RF, Resonance Frequency, WBA, Wideband Absorbance, YM, Amplitude, YA, Phase.

P1:MD vs. Control A; P2: LVAS vs. Control B; P3: MD vs. LVAS.

Bonferroni correction was applied to adjust for multiple WBA comparisons across 16 frequency points (αcorrected = 0.0031). Bonferroni correction was applied to adjust for multiple YM/YA comparisons across 4 frequency points (αcorrected = 0.0125). Significant values are highlighted in red.

### Resonant frequencies, admittance amplitude, and phase in adults with MD and LVAS

Participants with MD demonstrated significantly lower resonant frequencies compared to the control group (p < 0.05), with averages of 748.00 Hz (SD = 223.80) and 850.61 Hz (SD = 236.90), respectively. Additionally, the admittance amplitude in the MD group was higher at 226 Hz and 678 Hz (p < 0.05). However, there was no significant difference in phase between the MD and control groups (p > 0.05). Similarly, the LVAS group showed a significant decrease in resonant frequencies compared to the control group (p < 0.05). The admittance amplitude and phase angle in the LVAS group were no significantly difference compared to the control group (p > 0.05). When comparing the MD and LVAS groups, there were no significant differences in resonant frequencies (p > 0.05), but significant differences were observed in admittance amplitude at 226–1000 Hz and phase angle at 1000 Hz (p < 0.05). Shown in Table 3.

## Discussion

This study provides a retrospective analysis of WAI patterns in adult patients with MD and LVAS, revealing distinct biomechanical alterations in the middle ear due to the underlying inner ear abnormalities in these two conditions. Our findings indicate that the LVAS group exhibited significantly lower WBA at 1000–2520 Hz, while the MD group demonstrated reduced WBA primarily at 2000–4000 Hz, pointing to frequency-dependent disruptions in sound energy transfer caused by distinct pathophysiological mechanisms. The ROC values for distinguishing LVAS from MD and both from control groups suggest that WAI has potential as a diagnostic tool for differentiating these inner ear disorders. In both the MD and LVAS groups, resonant frequencies were significantly lower compared to the control group, reflecting changes in the stiffness and mass properties of the middle ear and cochlea.

### The Impact of Sensorineural Hearing Loss (SNHL) on WBA

Feeney et al. reported that SNHL is associated with elevated wideband Stapedius Reflex Thresholds (ART), particularly at 1 kHz, 2 kHz, and Broadband Noise (BBN) frequencies.^10^ However, these changes primarily affect WAI-related reflex thresholds rather than WBA itself. Merchant et al. further demonstrated that WBA effectively differentiates Conductive Hearing Loss (CHL) from normal hearing but does not show systematic alterations in SNHL patients.^11^ These findings suggest that, although hearing loss is a confounding factor in audiological assessments, WBA measurements remain reliable for assessing middle ear mechanics.

However, it is important to acknowledge that certain inner ear pathologies ‒ particularly third-window syndromes (e.g., Large Vestibular Aqueduct Syndrome [LVAS])^4^ — can introduce significant changes in WBA due to altered cochlear impedance and abnormal inner ear fluid dynamics. Unlike isolated SNHL, which primarily affects cochlear transduction without directly altering middle ear stiffness or mass, third-window pathologies create additional sound transmission pathways that modify WBA patterns.

Due to the limited number of available LVAS cases, we were unable to fully match hearing thresholds between groups. However, the inclusion of Supplement Table S1 ensures transparency regarding hearing loss distribution and allows for a more refined interpretation of WBA patterns. Future studies with strictly hearing-matched cohorts will be necessary to further isolate the effects of inner ear pathology on WBA.

### The role of vestibular aqueduct size

The differences in WBA patterns between LVAS and MD may be influenced by Vestibular Aqueduct (VA) size. While it is not definitively known whether VA size is a determining factor in all cases, literature reports indicate that a significant proportion of MD patients exhibit a smaller or non-visible VA, which may contribute to endolymphatic hydrops due to impaired fluid drainage and subsequent increased inner ear pressure.^12^, ^13^ This increased pressure has been reported to primarily affect mid- to high-frequency sound transmission, as documented in previous MD studies, which have shown WBA reductions in the mid- to high-frequency range, consistent with our findings.^14^, ^15^ The elevated pressure likely impacts the stiffness and mass properties of the middle ear, leading to lower resonant frequencies and higher admittance amplitudes.^4^, ^6^

In contrast, LVAS is characterized by an enlarged VA, confirmed by CT imaging in this study. The enlarged VA causes a third mobile window effect, allowing excessive fluid movement and pressure dissipation, which predominantly affects low- to mid-frequency sound transmission. This disruption in fluid dynamics accounts for the low-frequency WBA reductions observed in LVAS patients and is further reflected in lower admittance amplitudes and altered phase angles.^16^, ^17^ The third window effect is specific to conditions like LVAS, where an abnormal VA alters the acoustic impedance of the middle ear, particularly impacting sound transmission at lower frequencies.^18^

However, while the literature suggests that MD patients frequently have a smaller VA, this study did not include imaging evidence to directly confirm VA size in our MD cohort. The WBA results for MD, showing mid- to high-frequency reductions, are consistent with a smaller VA leading to fluid accumulation and endolymphatic hydrops, but this remains speculative without imaging confirmation. Thus, while a smaller VA may be a contributing factor in MD, further verification through imaging studies such as CT or MRI is necessary to confirm this hypothesis.

Given the absence of imaging data on VA size in MD in our study, it is important to interpret these findings with caution. While the observed WAI patterns in MD and LVAS suggest a possible relationship between VA size and sound transmission impairment, it cannot be definitively concluded that VA size alone accounts for these differences. Future studies with imaging confirmation are essential to explore the role of VA size in MD, and caution is warranted when attributing the observed pathophysiological differences between MD and LVAS solely to VA size without further evidence.

These findings highlight the complexity of inner ear pathophysiology and the importance of long-term follow-up in MD patients. Future studies incorporating CT or MRI imaging are needed to explore the role of vestibular aqueduct size in middle ear mechanics and its potential contribution to WBA changes.

### WAI and the third window mechanism

The concept of the third window mechanism is crucial for understanding the WAI patterns observed in LVAS. The third window refers to an abnormal fluid pathway within the inner ear, typically created by an enlarged VA or other structural anomalies. This additional window causes sound energy to dissipate, reducing the efficiency of sound transmission through the middle ear. In LVAS, the third window effect primarily impacts low-frequency sound transmission, as sound energy is shunted through the enlarged VA, leading to significant WBA reductions in this frequency range.^17^, ^18^

Furthermore, research suggests that third Mobile Window Abnormalities (TMWA) can alter middle ear mechanics beyond just the enlarged VA effect. LVAS can lead to abnormally high WBA at low frequencies and reduced WBA at middle frequencies, reflecting increased middle ear compliance due to TMWA.^4^ Unlike SNHL, which primarily affects cochlear function, TMWA directly modifies middle ear impedance. This distinction is critical when interpreting WBA results, as inner ear pressure-related abnormalities can confound middle ear assessments.

Although WAI can identify middle ear impedance changes in MD and LVAS, it should be used as a complementary tool rather than a substitute for structural imaging in definitive diagnosis. Instead, WAI may serve as an accessible, cost-effective screening tool that can facilitate early identification of patients who require further imaging or specialized evaluation. This approach may improve clinical efficiency by prioritizing diagnostic imaging for cases that demonstrate significant abnormalities in WAI profiles.

### Limitations

One of the primary limitations of this study is the challenge in accurately assessing the size of the vestibular aqueduct in the MD group from gadolinium-enhanced MRI. Future studies should employ high-resolution CT to provide more accurate measurements of VA size in MD patients and assess its impact on WBA patterns. Additionally, the small sample size in the LVAS group limits the statistical power of our findings and reduces the generalizability of the results. Another limitation is that while our results suggest that WBA is primarily influenced by inner ear pressure dynamics rather than SNHL, our study does not provide conclusive evidence that SNHL does not significantly alter WBA. Further studies with larger, hearing-matched cohorts are needed to clarify the potential impact of SNHL on WBA measurements. Furthermore, this study does not address potential longitudinal changes in WBA patterns over time. Larger studies with repeated WAI measurements could provide valuable insights into disease progression and its impact on middle ear mechanics.

## Conclusion

This study provides evidence that WAI is a valuable tool for distinguishing MD and LVAS from normal controls and has potential for guiding early clinical evaluation. The distinct WBA patterns observed suggest that VA size and inner ear pressure alterations contribute to these differences, making WAI a potential functional marker for these conditions. However, a comprehensive diagnostic approach combining WAI, audiometric evaluations, and imaging remains essential for accurate differentiation of inner ear disorders.

## ORCID ID

Yi Um: 0009-0004-7699-8345

Huan Lin: 0003-3536-478X

Chanfeng Shen: 0009-0005-2484-5221

Huiying Zhang: 0009-0005-1893-4068

Fei Zhao: 0000-0003-1841-4466

Yuehua Qiao: 0000-0002-6485-4940

Wen Liu: 0000-0002-3984-1216

## CRediT authorship contribution statement

WJ, YM, and HL contributed to the study design, data collection, analysis, and drafting of the manuscript. YHQ, XYL, and WL conceptualized the project and provided input on the study design. CFS and HYZ assisted with data collection and analysis. ZF was responsible for critical manuscript review. All authors participated in the review, provided substantial revisions, and approved the final version of the manuscript.

## Funding

This work was supported by the Jiangsu postdoctoral research funding program (1701063B), the medical science and technology innovation project of Xuzhou Municipal Health Commission (XWKYHT20220149); the Open project of key laboratories in colleges and universities in Jiangsu Province (XZSYSKF2022005) and Postgraduate Research & Practice Innovation Program of Jiangsu Province (KYCX23_2940).

## Declaration of competing interest

The authors declare that they have no known competing financial interests or personal relationships that could have appeared to influence the work reported in this paper.
