# Supplementary material for: Effects of inner ear abnormalities on middle ear mechanics: Findings from adults with MD and LVAS
Source: Braz J Otorhinolaryngol. 2025 Sep 27;92(1):101673. doi: 10.1016/j.bjorl.2025.101673 (PMC12510180; doi:10.1016/j.bjorl.2025.101673)
Supplement: Supplementary file 1 [file mmc1.docx]

**BJORL-D-25-00021**_**Supplement Material**

**Supplement Table 1** Demographic information and audiological characteristics of the adults with MD and LVAS.

| **Case** | **Age (Yrs)** | **Gender** | **Syndrome ear side** | **Hearing Classification^a^** | **G-MRI (EH ear side) / CT (Enlarged ear side)** |
| --- | --- | --- | --- | --- | --- |
| MD1 | 56 | Male | R | II | R |
| MD2 | 48 | Female | L | IV | L-Mild |
| MD3 | 29 | Female | L | I | R-Mild |
| MD4 | 33 | Female | L | II | L |
| MD5 | 48 | Male | L | III | L |
| MD6 | 33 | Female | L | III | L |
| MD7 | 26 | Female | L | II | L |
| MD8 | 33 | Female | R | II | R |
| MD9 | 34 | Female | L | IV | L |
| MD10 | 31 | Male | L | IV | L |
| MD11 | 31 | Male | R | IV | R |
| MD12 | 49 | Male | L | III | L |
| MD13 | 49 | Male | R | III | R |
| MD14 | 59 | Male | R | III | R |
| MD15 | 68 | Male | L | IV | L |
| MD16 | 37 | Male | R | IV | R |
| MD17 | 32 | Male | L | III | L |
| MD18 | 77 | Female | R | IV | R |
| MD19 | 35 | Female | L | IV | L |
| MD20 | 35 | Female | R | I | R-Mild |
| MD21 | 43 | Male | R | I | R |
| MD22 | 55 | Male | R | II | R |
| MD23 | 41 | Female | R | II | R |
| MD24 | 43 | Female | L | I | L-Mild |
| MD25 | 45 | Female | R | I | R |
| MD26 | 45 | Female | L | III | L-Mild |
| MD27 | 43 | Male | L | III | L |
| MD28 | 48 | Female | L | III | L |
| MD29 | 48 | Female | R | I | R |
| MD30 | 49 | Female | L | IV | L |
| MD31 | 49 | Male | L | II | Bilateral-L |
| MD32 | 49 | Male | R | II | Bilateral-R |
| MD33 | 68 | Male | R | IV | R-Mild |
| MD34 | 51 | Female | R | II | R |
| MD35 | 53 | Female | R | III | R |
| MD36 | 53 | Female | R | III | R-Mild |
| MD37 | 61 | Male | L | I | Bilateral-L |
| MD38 | 21 | Male | L | III | L |
| MD39 | 21 | Male | R | IV | R |
| MD40 | 28 | Male | L | III | L |
| MD41 | 25 | Male | L | I | L-Mild |
| LVAS1 | 20 | Male | L | IV | L |
| LVAS2 | 20 | Male | R | IV | R |
| LVAS3 | 26 | Male | L | IV | L |
| LVAS4 | 26 | Male | R | IV | R |
| LVAS5 | 28 | Male | L | IV | L |
| LVAS6 | 28 | Male | R | IV | R |
| LVAS7 | 30 | Male | R | IV | R |
| LVAS8 | 30 | Male | L | IV | L |
| LVAS9 | 21 | Male | L | IV | L |
| LVAS10 | 21 | Male | R | IV | R |
| LVAS11 | 35 | Female | L | IV | L |
| LVAS12 | 35 | Female | R | IV | R |
| LVAS13 | 28 | Male | L | IV | L |
| LVAS14 | 28 | Male | R | IV | R |
| LVAS15 | 32 | Male | L | IV | L |
| LVAS16 | 32 | Male | R | IV | R |
| LVAS17 | 27 | Male | L | IV | L |
| LVAS18 | 27 | Male | R | IV | R |

^a^ Hearing Classification Criteria (average hearing threshold of 500, 1000, 2000, 4000 Hz by air conduction): I: 25 dB HL, II: 26‒40 dB HL, III: 41‒70 dB HL, IV: >70 dB HL.
